# Unbiased Thiol-Labeling and Top-Down Proteomic Analyses Implicate Multiple Proteins in the Late Steps of Regulated Secretion

**DOI:** 10.3390/proteomes7040034

**Published:** 2019-09-27

**Authors:** Kendra L. Furber, Peter S. Backlund, Alfred L. Yergey, Jens R. Coorssen

**Affiliations:** 1 Northern Medical Program, University of Northern British Columbia, Prince George, BC V2N 4Z9, Canada; 2 Eunice Kennedy Shriver National Institute of Child Health and Human Development, National Institutes of Health, Bethesda, MD 20892, USA; 3 Department of Health Sciences, Faculty of Applied Health Sciences and Department of Biological Sciences, Faculty of Mathematics & Science, Brock University, St. Catharines, ON L2S 3A1, Canada

**Keywords:** exocytosis, calcium, secretory vesicle, thiol-reactivity, 2-dimensional gel electrophoresis, Rab GTPase

## Abstract

Regulated exocytosis enables temporal and spatial control over the secretion of biologically active compounds; however, the mechanism by which Ca^2+^ modulates different stages of exocytosis is still poorly understood. For an unbiased, top-down proteomic approach, select thiol- reactive reagents were used to investigate this process in release-ready native secretory vesicles. We previously characterized a biphasic effect of these reagents on Ca^2+^-triggered exocytosis: low doses potentiated Ca^2+^ sensitivity, whereas high doses inhibited Ca^2+^ sensitivity and extent of vesicle fusion. Capitalizing on this novel potentiating effect, we have now identified fluorescent thiol- reactive reagents producing the same effects: Lucifer yellow iodoacetamide, monobromobimane, and dibromobimane. Top-down proteomic analyses of fluorescently labeled proteins from total and cholesterol-enriched vesicle membrane fractions using two-dimensional gel electrophoresis coupled with mass spectrometry identified several candidate targets, some of which have been previously linked to the late steps of regulated exocytosis and some of which are novel. Initial validation studies indicate that Rab proteins are involved in the modulation of Ca^2+^ sensitivity, and thus the efficiency of membrane fusion, which may, in part, be linked to their previously identified upstream roles in vesicle docking.

## 1. Introduction

Regulated exocytosis is a fundamental process for the release of bioactive molecules from cells. Several discrete stages are involved in the exocytotic pathway, including trafficking of secretory vesicles containing cellular cargo to the appropriate target membrane, tethering and docking for attachment of secretory vesicles to release sites, priming of the vesicles to render them fusion competent, Ca^2+^ sensing and triggering events to regulate and facilitate fusion of the secretory vesicle and plasma membranes (PM), and thus merger of the two lipid bilayers to form a fusion pore releasing vesicular contents. This involves the coordinated actions of both lipid and protein components of the secretory vesicle membrane, particularly the later steps of Ca^2+^-triggered exocytosis. There have been recent advances in understanding how the lipid matrix may modulate upstream docking/priming steps [1,2,3,4,5,6,7], regulate the Ca^2+^ sensitivity and efficiency of fusion [5,6,7,8,9,10,11], and facilitate or impede the merger of biological membranes [1,6,7,9,12,13,14]. However, there remains debate regarding the identity of proteins involved in the Ca^2+^-sensing and triggering steps that regulate the efficiency of exocytosis.

The molecular mechanisms underlying regulated exocytosis are highly conserved across numerous secretory cell types, including neurons, endocrine and exocrine cells, immune cells and oocytes [15]. The lipid and protein composition of secretory vesicles are, thus, similar across various cell types [16,17,18,19]. Homologues of several proteins involved in membrane trafficking pathways are present in species ranging from unicellular organisms to humans [20,21,22]. Thiol-reactive reagents, such as N- ethylmaleimide (NEM), have historically been shown to inhibit membrane trafficking events, which subsequently led to the discovery of NEM-sensitive factor (NSF) [23], soluble NSF attachment protein (α-SNAP) [24] and SNAP receptors (SNAREs) [25]. Members of the SNARE family (syntaxin, SNAP-25 and VAMP), along with interaction partners, such as Sec1/MUNC (SM) proteins, are proposed to be key players in the late steps of exocytosis [26]. A more recent study also employed NEM to further elucidate the intricate roles of NSF, MUNC-18 and MUNC-13 in regulating SNARE complex assembly during priming and de-priming steps [27]. Among other proteins enriched on secretory vesicles, Rab GTPases and synapotagmins have been implicated in tethering/docking and Ca^2+^ sensing, respectively [26]. While these secretory vesicle proteins all have critical roles in the exocytotic pathway, the exact nature of their function(s) at each stage remains unknown [28,29,30,31,32].

Isolated secretory vesicles—cortical vesicles (CVs) from sea urchin oocytes—provide a well characterized model for investigating the fundamental molecular machinery involved in regulated exocytosis in native membranes [22,33,34]. Isolated CVs undergo homotypic fusion through the same conserved mechanism underlying CV–PM fusion, indicating that the vesicles contain the minimal essential components for docking/priming, Ca^2+^-sensing/triggering and membrane merging [35] Interfering with the proper function of native proteins on CVs, through non-selective degradation or chemical modification, enables the dissection of functions in the late steps of exocytosis [30,31,32,35,36,37,38]. As in other model systems, NEM inhibits the Ca^2+^-sensitivity, rate and extent of CV–PM [37,38] and CV–CV [30,35] fusion, which is independent of NSF [36]. Previously, we characterized a novel effect of thiol reagents—an enhancement of the Ca^2+^-sensitivity and rate of CV–PM and CV–CV fusion after treatment with iodoacetamide (IA) [39]. Further investigation determined that hydrophobic and hydrophilic reagents had a greater propensity for inhibiting or potentiating Ca^2+^-triggered membrane fusion, respectively [40] (for structures of thiol-reactive reagents, see Figure S1). This suggested, in agreement with studies using Ca^2+^ mimetics [30], that multiple proteins work in cooperation to modulate the efficiency of regulated exocytosis.

Here, we employ fluorescent thiol-reactive reagents in an un-biased, top-down proteomic approach to identify proteoforms that modulate the efficiency of Ca^2+^-triggered exocytosis. These reagents can be used to tightly couple sensitive functional and molecular analyses of native secretory vesicles (i.e., CVs), simultaneously enhancing Ca^2+^-sensitivity while labeling select components of the membrane proteome. We have previously shown the feasibility of this approach with the fluorescent thiol-reagent, Lucifer yellow iodoacetamide (LYIA) [40]. As thiol-reactive reagents are non-selective, numerous ‘background’ proteins become labeled. Thus, to narrow the list of candidate proteins, multiple fluorescent thiol reagents were characterized for their ability to potentiate fusion. LYIA, along with monobromobimane (mBB) and dibromobimane (dBB), were used as functional tags to identify a common set of proteoforms involved in the Ca^2+^ sensing steps of regulated membrane fusion. As a well-established top-down approach, labeled proteoforms were analyzed by high- resolution, two-dimensional gel electrophoresis (2DE) followed by mass spectrometric analysis of tryptic spot digests for protein identification (i.e., MALDI-TOF/TOF or LC/MS/MS) [41,42,43,44]. Considering the evidence for lipid microdomain involvement in the exocytotic mechanism [8,9,10,11,45,46], labeled proteoforms from both total CV membrane and cholesterol-enriched CV membrane isolates were resolved by 2DE. Protein spots labeled by all three reagents, and detected in cholesterol- enriched membranes, included several Rab GTPases. Further investigation indicated that Rab proteins may act in multiple steps to modulate the efficiency of exocytosis. Other identified proteoforms will be interesting subjects for further investigation, particularly as some may provide critical links between lipid and protein contributions to the late steps of regulated exocytosis.

## 2. Materials and Methods

### 2.1. CV Isolation and Fusion Assays

The isolation of CVs from unfertilized oocytes of *Strongylocentrotus purpuratus* (Westwind, Victoria, BC, Canada) was performed as previously described [3,6,9,10,11,12,39,40]. For thiol treatments, isolated CVs were suspended in baseline intracellular media (BIM; 210 mM potassium glutamate, 500 mM glycine, 10 mM NaCl, 10 mM Pipes, 0.05 mM CaCl_2_, 1 mM MgCl_2_, 1 mM EGTA, pH 6.7) supplemented with 2.5 mM ATP and broad-spectrum protease inhibitors. Stock solutions of fluorescent thiol reagents (Molecular Probes; ThermoFisher Scientific) were made in dimethyl sulfoxide (DMSO) or dimethylformamide (DMF). Treatments were delivered by spiking a small volume of stock solution directly into CV suspensions of optical density (OD) = 1.0–1.1. Final solvent concentrations were ≤1% and showed no effect on fusion parameters. Free-floating CVs were incubated with thiol reagents for 20 min at 25 °C, then centrifuged at 2000× *g* to wash out excess reagent. Samples were suspended in fresh BIM, then aliquoted for functional and molecular analyses. Aliquots for functional assays were diluted to a working OD = 0.30–0.35. Aliquots for molecular analyses were centrifuged (2000× *g*) and the pellet stored at −80 °C until further analyses.

For analysis of GTP and Rab GTPase function, isolated CVs were suspended in BIM (pH 6.7) supplemented with protease inhibitor cocktail without ATP. GTP or GTPγS were added to CV suspensions of OD 0.30–0.35 to the final concentrations indicated. The free-floating CV suspensions were allowed to equilibrate for 10 min on ice, then immediately assayed for fusion parameters in the presence of the nucleotide. A RAB peptide, including a portion of the effector binding domain (rab3 GTPase; NP_001116967 a.a. 52-67 (vstvgidfkvktvfrq) [47]), and a scrambled (SC) peptide (vfvdvtkqvsgrftik) were commercially synthesized (Biomatik, Cambridge, ON, Canada). Peptides were added to CV suspensions of OD 0.30–0.35 to the final concentrations indicated and supplemented with 100 µM GTPγS. The free-floating CV suspensions were incubated for 30 min at 25 °C, then immediately assayed for fusion parameters in the presence of the peptide. For thiol pre- treatments, a stock solution of IA (Sigma) in water was delivered to CV suspensions of OD = 1.0–1.1 and incubated for 30 min at 25 °C. To washout excess reagent, CVs were centrifuged at 2000× *g* and suspended in fresh BIM prior to the addition of GTPγS and peptides.

CV–CV fusion assays were carried out at room temperature using a straightforward light-scattering paradigm, in which the ∆OD observed upon hydration and dispersal of CV contents were used to quantify the extent of fusion [33,35,37]. For standard endpoint and kinetic assays, free-floating vesicles were brought into contact at the bottom of multi-well plates by low speed centrifugation to bypass upstream stages of exocytosis, ensuring optimal inter-membrane contact before the addition of Ca^2+^ or Sr^2+^ [3,6,9,10,11,12,30,39]. In some cases, a modified endpoint assay in which vesicles were allowed to settle into contact was also used to assess the capacity of the native vesicular machinery for tethering/docking/priming [3,6,30,39]. Fusion data for both endpoint and kinetic assays were normalized to control conditions, with the ∆OD at the low and high [Ca^2+^]_free_ plateaus defining 0% and 100% fusion, respectively; Sr^2+^ data were normalized to control Ca^2+^ data determined in parallel, as previously described [30,39]. Final [Ca^2+^]_free_ were measured with a Ca^2+^ sensitive electrode (World Precision Instruments, Sarasota, FL) and final [Sr^2+^]_free_ were calculated using MaxChelator (WINMAXC32 v2.50). Endpoint activity curves were fit using a log-normal cumulative function to determine the extent, slope and Ca^2+^-sensitivity of fusion [48]. Control conditions were fit to a 2-parameter model (with fusion extent set to 100%, by definition) and treatment conditions were fit to a 3-parameter model using TableCurve 2D (v5.0, SYSTAT, Richmond, CA). For each experiment (n), all conditions were tested in technical replicates of 3–6. Data are reported as mean ± SEM; one way ANOVA and Bonferroni post-hoc analyses were carried out to identify significant differences compared to controls.

### 2.2. Proteome Resolution by 2DE

The isolation of total CV membrane [49] and cholesterol-enriched CV membrane [45] were performed as previously described. In brief, CV pellets were lysed in hypotonic buffer (10 mM PIPES; pH 6.7) supplemented with 5 mM tris(2-carboxyethyl) phosphine (TCEP), 5 mM IA and broad-spectrum protease, kinase and phosphatase inhibitors [49,50]. Membrane fragments were isolated by centrifugation at 120,000× *g*. Cholesterol-enriched membrane fragments were further fractionated by density centrifugation on sucrose gradients (40%/30%/22%/10% sucrose, 25 mM Tris; pH 7), collected from the 22%/10% interface, washed in 25 mM Tris buffer and then isolated by centrifugation at 120,000× *g*. Membrane pellets were solubilized in 2DE sample buffer (8 M urea, 2 M thiourea, 4% CHAPS), supplemented with the broad-spectrum protease inhibitor cocktail, and protein concentration was determined using the EZQ Protein Quantitation Kit (ThermoFisher). Membrane proteins were resolved by well-established, optimized 2DE protocols, with slight modifications [2,8,40,50]. In the first dimension, proteins were resolved on immobilized nonlinear pH 3–10 gradients (3–10 NL IPG; Bio-Rad). Prior to loading, samples were reduced with 5 mM TCEP for 1 h, followed by alkylation with 230 mM acrylamide for 1 h at 25 °C. A total of 100 µg or 300 µg protein was loaded onto 7 cm (mini format) or 17 cm (large format) IPG strips, respectively, by passive rehydration for 12–14 h at 25 °C. Isoelectric focusing (IEF) was carried out at 4000 V for a total of 37,500 Vh across 7 cm IPG, or at 10,000 V for a total of 75,000 Vh for 17 cm IPG at 17 °C; voltage was ramped to 250 for 15 min, and then ramped linearly for 2 h at 50 µA/gel. IPG strips were treated in equilibration buffer (6 M urea, 2% SDS (*w*/*v*), 20% glycerol (w/v), 375 mM Tris; pH 8.8) supplemented with 5 mM TCEP for 10 min, followed by equilibration buffer supplemented with 350 mM acrylamide for 10 min. The second-dimension resolution was in large format SDS-PAGE gradient gels (10–14% T; 3.6% C) at a constant current of 100 mA. To improve the detection of thiol-labels, proteomes were electro-transferred to 0.2 µm polyvinylidenefluoride (PVDF) membrane (Millipore-Sigma or GE Life Sciences) using modified Towbin buffer at a constant current of 50 mA for 14 h at 4 °C [40]. Parallel samples were resolved for the detection of fluorescent thiol-labelled proteins (excitation/emission: LYIA 450/530, mBB 390/530 and dBB 390/530) on PVDF membranes, and total protein (ex/em: SyproRuby 480/620; Bio-Rad) in SDS-PAGE gels. Images were acquired using the ProXpress Proteomic Imaging System (Perkin-Elmer, Boston MA) and analyzed with Delta2D (Decodon, Greifswald, Germany). Following spot matching and warping, gel images for each experimental group were merged pixel-by-pixel, yielding an average proteome map.

### 2.3. Mass Spectrometry

For mass spectrometric analyses, spots of interest were excised from 2DE gels by visualization under a UV light source. Proteins underwent in-gel digestion, as previously described [51,52]. Briefly, gel pieces were dried in a vacuum centrifuge and then incubated with 200 ng of modified trypsin (Promega, Madison, WI) in 100 mM ammonium bicarbonate overnight at 37 °C. The peptides were extracted from the gel pieces using 5% formic acid: acetonitrile (1:1, v/v) followed by a second extraction with 5% formic acid: acetonitrile (5:95, *v*/*v*). The peptide extracts were vacuum-dried, solubilized in 0.1% trifluoroacetic acid (TFA) and cleaned using C18 ZipTips (Millipore, Billerica, MA). The resulting peptides were analyzed by MALDI-TOF/TOF using an ABI 4800 Proteomics Analyzer (Applied Biosystems, Foster City, CA) and/or by LC-ESI/MS/MS using an LCQ DECA ion trap mass spectrometer (ThermoFisher, Waltham, MA).

MS and MS/MS spectra from both the MALDI and LC-ESI measurements were then analyzed. Both b and y ion series were used to match to protein sequences present in the “other metazoa” subset (5,474,206 sequences) of NCBIprot database (29 April 2018) using the Mascot v 2.2 (Matrix Science, Boston, MA) search program. Parameters for the database search with Mascot MS/MS Ions Search software were set as follows: enzyme, trypsin; one missed tryptic cleavage site permitted; fixed modification, carbamidomethylation of cysteine; variable modification, methionine oxidation. For MALDI TOF-TOF data, mass tolerance for precursor ions was set at ±0.15 Da and mass tolerance for fragment ions ±0.06 Da. The instrument was mass calibrated by the plate model method using ABI peptide standards applied to the calibration wells; mass calibration was verified using the masses of trypsin autolysis peptides in each sample or from spectra of known peptide standards spotted in nearby wells to the sample spots. For LC/ESI ion trap data, precursor ion ±1.2 Da and fragment ion ±0.6 Da were used. The cut-off for peptide identification was set at greater than 95.0% probability and protein identification at greater than 99.0% probability. Protein identifications based on a minimum of 2 fragmented peptides are reported.

## 3. Results

### 3.1. Fluorescent Thiol-Reactive Reagents Modulate the Ca^2+^ Sensitivity and Rate of Membrane fusion

**Figure 1:**
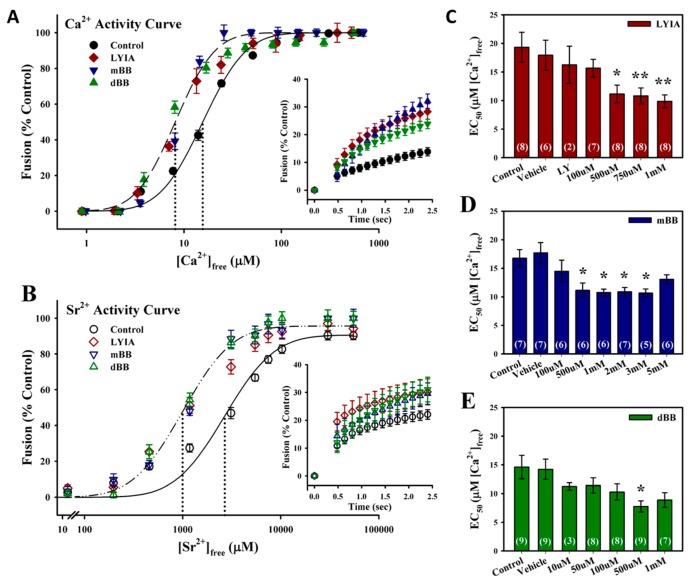
Promotion of the Ca^2+^-triggered steps of exocytosis with fluorescent thiol reagents. (**A**) Ca^2+^ activity curves (*n* = 7–9) for cortical vesicle (CV)–CV fusion after 20 min treatment with 750 µM LYIA, 2 mM mBB or 500 µM dBB. Fusion kinetics in response to 24.3 ± 2.2 µM [Ca^2+^]_free_ (inset; *n* = 6–8). (**B**) Sr^2+^ activity curves (*n* = 6 or 7) for CV–CV fusion after 20 min treatment with 750 µM LYIA, 2 mM mBB or 500 µM dBB. Fusion kinetics in response to 3110 µM [Sr^2+^]_free_ (inset; *n* = 5 or 6). (**C**–**E**) Summary of concentration-dependent effects of LYIA (**C**), mBB (**D**) and dBB (**E**) on the Ca^2+^-sensitivity of CV–CV fusion. Data presented as mean ± SEM; statistical analysis by one-way ANOVA with Bonferroni multiple comparison test versus control (* *p*< 0.05, ** *p*< 0.01, *** *p* < 0.001).

Ca^2+^ activity curves for homotypic CV–CV fusion (*n* = 24) yielded a typical sigmodal shape [48] with an EC_50_ of 16.3 ± 1.3 µM [Ca^2+^]_free_ (Figure 1A). The thiol reagents used have a biphasic effect, with low concentrations potentiating fusion parameters and high concentrations inhibiting all parameters of Ca^2+^-triggered membrane fusion [39,40]. Three fluorescent thiol reagents were found to enhance the efficiency of fusion at low concentrations with short incubation times. Treatment of CV with 750 µM LYIA (*n* = 8), 2 mM mBB (*n* = 7) and 500 µM dBB (*n* = 9) for 20 min resulted in leftward shifts in Ca^2+^ sensitivity to respective EC_50_’s of 10.8 ± 1.4 µM, 10.9 ± 0.7 µM and 7.8 ± 1.0 µM [Ca^2+^]_free_ (Figure 1A). An enhancement of late kinetics in response to low [Ca^2+^]_free_ = 24.3 ± 2.2 µM was also observed with 750 µM LYIA (*n* = 7), 2 mM mBB (*n* = 6) and 500 µM dBB (*n* = 8) treatments (Figure 1A, inset). To further elucidate the involvement of thiol sites in modulating the Ca^2+^ sensitivity of triggered exocytosis, a weak Ca^2+^ mimetic was used [30,39]. The resulting Sr^2+^ activity curves had a similar sigmodal shape to the Ca^2+^ activity curves; however, Sr^2+^ is much less efficient at triggering CV–CV fusion, requiring 100-fold higher concentrations. Thus, Sr^2+^ activity curves from untreated CV (*n* = 12) had an EC_50_ of 2600.6 ± 374.8 µM [Sr^2+^]_free_ (Figure 1B). Treatment of CV with 750 µM LYIA (*n* = 7), 2 mM mBB (*n* = 6) and 500 µM dBB (*n* = 7) for 20 min resulted in leftward shifts in Sr^2+^ sensitivity to EC_50_’s of 1222.2 ± 334.3 µM, 1148.7 ± 89.4 µM and 1072.8 ± 159.8 µM [Sr^2+^]_free_, respectively (Figure 1B). There was also an enhancement of initial kinetics with 750 µM LYIA treatment (*n* = 4), and late kinetics with 2 mM mBB (*n* = 4) and 500 µM dBB (*n* = 6) treatments, in response to low [Sr^2+^]_free_ = 3110 µM (Figure 1B, inset). The potentiation of the Ca^2+^-sensitivity of CV–CV fusion was observed for these fluorescent thiol reagents across treatments in the high micromolar to low millimolar range (Figure 1C,D), while higher treatment concentrations resulted in the inhibition of the Ca^2+^-sensitivity and extent of fusion (Figure S2).

### 3.2. Identification of Fluorescently Labeled Candidate Proteoforms

**Figure 2:**
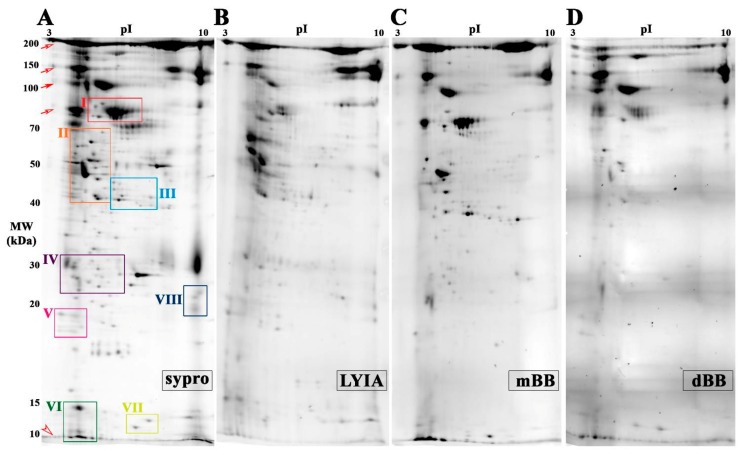
Total membrane proteome of CV treated with fluorescent thiol reagents. (**A**) Average two-dimensional gel electrophoresis (2DE) gel image of total membrane proteome from untreated CV resolved by mini 3–10 NL IPG and large 10%–14% SDS-PAGE format, stained for total protein with Sypro Ruby (*n* = 10). The average PVDF blot images (*n* = 4) scanned for LYIA (**B**), mBB (**C**) and dBB (**D**); addition of negatively charged LYIA shifts pIs of proteoforms relative to other labels. Open and closed arrows indicate background labeling of CV proteins that are normally released to form the fertilization envelope. Open arrow head indicates low abundance, poorly resolved peptides co-migrating at the gel front. Boxed regions of interest contain spots labeled by all three fluorescent reagents are shown in greater detail in Figure 3.

**Figure 3:**
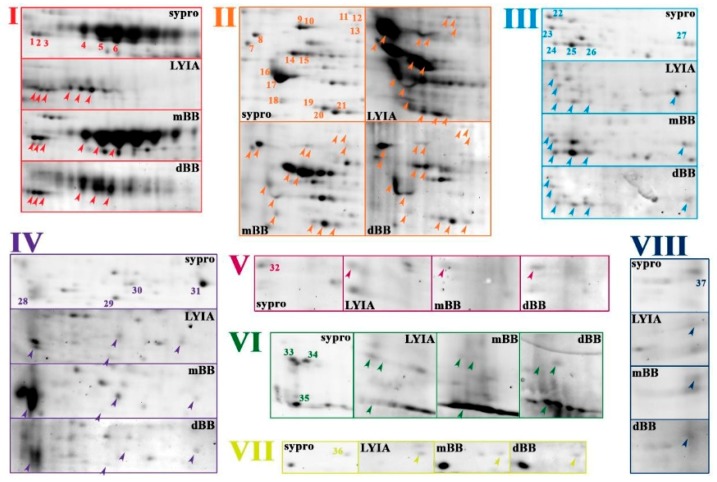
Fluorescent thiol-labeled spots excised from total membrane proteome.To improve resolution of protein spots, pooled CV membrane protein samples for each condition were resolved in duplicate by large format 2DE (i.e., 17 cm 3–10 NL IPG and large 10%–14% SDS-PAGE). A montage of boxed regions of interest indicated in Figure 2 is shown from representative gel image of untreated CV stained for total protein (Sypro ruby) and representative PVDF blot images for LYIA, mBB and dBB; addition of negatively charged LYIA shifts pIs of proteoforms relative to other labels. Protein spots reproducibly labeled with LYIA, mBB and dBB in both mini and large formats, as indicated by numbers in total protein images and arrowheads in thiol-labelled images, were excised for identification. The contrast level on each panel has been adjusted to visualize low-abundance-labeled spots; for full blot images refer to Figure 2.

To focus on proteoforms potentially involved in regulating the efficiency of Ca^2+^-triggered membrane fusion, CVs were exposed to thiol treatments and then subjected to molecular analyses to identify fluorescently labeled species. Proteoforms from both total and cholesterol-enriched CV membrane isolates were resolved by 2DE. The total CV membrane proteome resolved on mini-format 7 cm IPG strips revealed eight regions of interest having spots that were labeled by all three reagents with 100% reproducibility across all trials (*n* = 4 individual experiments per thiol reagent) (Figure 2 and Figure 3). In addition, a number of labeled spots were present in the low molecular weight peptide front were labeled (Figure 2; open arrow head). Strong labeling was also observed across several high abundance, high molecular weight streaks (Figure 2; open and closed arrows), which correspond to similar spot patterns in CV content proteomes (Figure S3). To better resolve labeled proteoforms for identification, equal amounts of remaining samples for each fluorescent thiol regent (ie., 75 µg protein) were pooled and resolved on large-format 17 cm IPG strips in the first dimension (*n* = 2 replicates per thiol reagent). In the eight boxed regions on the resulting 2D gels, a total of thirty-seven protein spots were observed that were labeled with LYIA, mBB and dBB (Figure 3). These spots were excised and analyzed by MALDI-TOF/TOF (Table 1).

**Figure 4:**
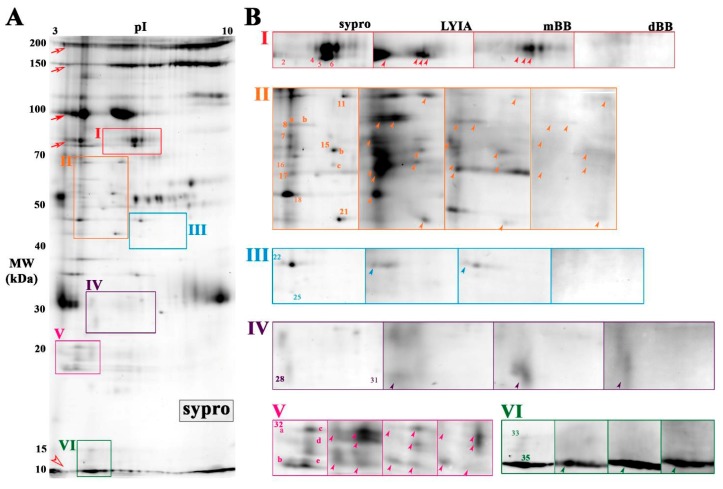
Cholesterol-enriched membrane proteome of CV treated with fluorescent thiol reagents. (**A**) Average 2DE gel image of cholesterol-enriched membrane proteome from untreated CV resolved on mini 3-10 NL IPG and large 10%–14% SDS-PAGE, stained with Sypro Ruby (*n* = 6). Boxed regions correspond to the same regions of interest shown for total membrane proteome. (**B**) A montage of boxed regions is shown from average gel image of untreated CV stained for total protein (Sypro ruby; *n* = 6) and average PVDF blot images (*n* = 3) scanned for LYIA, mBB and dBB. Protein spots corresponding to labeled proteins from the total membrane proteome are numbered accordingly. Open and closed arrows indicate background labeling of CV content proteins that are normally released to form the fertilization envelope. An open arrow head indicates labeled peptides co-migrating at the dye front. Protein spots reproducibly labeled with LYIA, mBB and dBB, as indicated by bold numbers in the total protein image and arrowheads in thiol-labelled images, were excised for identification. The contrast level on each panel has been adjusted to visualize low-abundance-labeled spots; for full blot images refer to Figure S4.

To further narrow the number of candidate proteoforms of interest, the isolation of cholesterol- enriched membrane fragments was used as a prefractionation tool. Cholesterol- and sphingomyelin-enriched domains regulate the efficiency of exocytosis by organizing critical components near the fusion site [8,9,10,11,45,46]. To this end, cholesterol was used as ‘bait’ to enrich proteoforms involved in regulated exocytosis, both to reduce the complexity of the proteome and improve the detection of low abundance labeled proteoforms [40,45]. The cholesterol-enriched CV membrane proteome (Figure 4; Figure S4) contained nineteen spots that corresponded to similar pI-and-MW as labeled spots from total CV membrane (Figure 3). Selective enrichment of certain proteoforms occurred in the cholesterol-enriched membrane proteome (Figure S5). For example, there was an apparent decrease in high molecular weight streaking (Figure 4A; open and closed arrows) whereas there was an apparent increase in the peptides that co-migrate at the gel front (Figure 4A; open arrow head). In Box V, one spot (#32) was reproducibly detected with total protein stain Sypro Ruby in the CV membrane proteome (Figure 2 and Figure 3), while several spots (#32a–e) were reproducibly detected in the cholesterol-enriched CV membrane proteome (Figure 4). In box II, there were two areas that appeared to have altered patterns of labeled spots (#8 and #15) compared to the total CV membrane proteome (Figure 2 and Figure 3). Of these, a total of thirteen spots were observed to be labeled by all three reagents with 100% reproducibility across all trials (*n* = 3 individual experiments per thiol reagent) (Figure 4B; labeled in bold). These spots were excised and analyzed by MALDI-TOF/TOF and/or LC-MS/MS (Table 1).

Mascot searches of the NCBIprot database resulted in the identification of the highest abundance protein species in thirty of the thirty-seven spots from the total CV membrane proteome, yielding up to a total of twenty-seven unique proteins that were reproducibly labeled. Many of these were also identified from corresponding spots (i.e., comparable pIs and MWs) in the cholesterol-enriched membrane proteomes, plus an additional eleven spots identified from the latter. All proteoforms identified (Table 1) contain at least one cysteine residue which may be modified; however, it may be the case that labeled and un-labeled proteoforms co-migrated. The proteoforms identified can be divided into seven broad canonical categories based on known biological function: metabolic processes, protein biosynthesis and folding, signal transduction, ion transport, cytoskeletal organization, vesicular transport and uncharacterized. Several gel plugs taken from the low molecular weight peptide front (Figure 2 and Figure 4; open arrowhead) did not yield protein identifications. A majority of the thiol-labeled proteoforms identified have defined functions in cellular metabolism, and these were initially considered to be less likely as candidates that may potentially modulate the Ca^2+^-sensitivity of regulated exocytosis. Among the most likely candidates were cytoskeletal proteins and Rab GTPases, which have both previously been implicated in vesicular trafficking. Several Rab GTPases were not only consistently labeled by all three fluorescent thiol reagents, but were also isolated in the cholesterol-enriched membrane proteome. We, therefore, chose to test these low MW G-proteins in a series of validation trials using the well-established CV–CV fusion assays.

### 3.3. Rab-GTPases Modulate the Efficiency of Exocytosis

**Figure 5:**
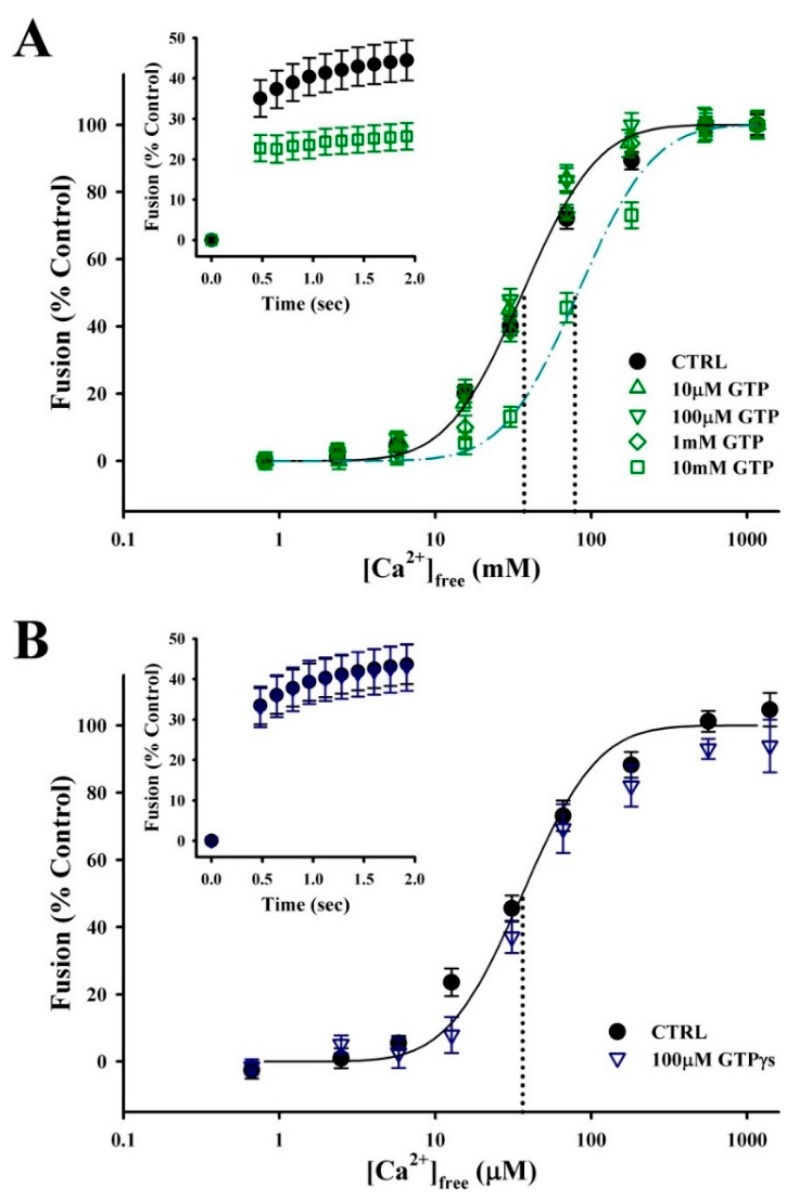
GTP has minimal impact on the efficiency of Ca^2+^-triggered exocytosis. (**A**) Ca^2+^ activity curves for standard CV–CV fusion assays supplemented with GTP (*n* = 5–6) and fusion kinetics in response to 33.7 ± 2.7 µM [Ca^2+^]_free_ (inset; *n* = 3). (**B**) Ca^2+^ activity curves for standard CV–CV fusion assay supplemented with GTPγS (*n* = 4) and fusion kinetics in response to 33.7 ± 2.7 µM [Ca^2+^]_free_ (inset; *n* = 3).

Synergistic effects between Ca^2+^ and GTP have been observed in different models of regulated secretion. While the only requirement for CV–CV fusion is an increase in [Ca^2+^]_free_ [53], GTP may modulate the efficiency of exocytosis. To explore this possibility, BIM media (without ATP) was supplemented with GTP or GTPγs immediately prior to fusion assays. While low concentrations had no effect on fusion, high concentrations of the nucleotide decreased the Ca^2+^ sensitivity and rate of CV–CV fusion (Figure 5A). Treatment with 10 mM GTP resulted in a rightward shift in EC_50_ from 37.1 ± 4.4 µm [Ca^2+^]_free_ under control conditions to 78.6 ± 13.3 µm [Ca^2+^]_free_ (*n* = 6; *p* ≤ 0.01). Initial fusion kinetics were also inhibited in response to 33.7 ± 2.7 µm [Ca^2+^]_free_ (*n* = 3; *p* ≤ 0.05), but not at higher [Ca^2+^]_free_. The addition of a nonhydrolyzable analogue GTPγS did not reduce the concentration of nucleotide required to inhibit fusion (*n* = 4) (Figure 5B). Furthermore, lengthening the incubation time to 30 min at 25 °C with GTP or GTPγS did not increase the potency of inhibition (data not shown).

**Figure 6:**
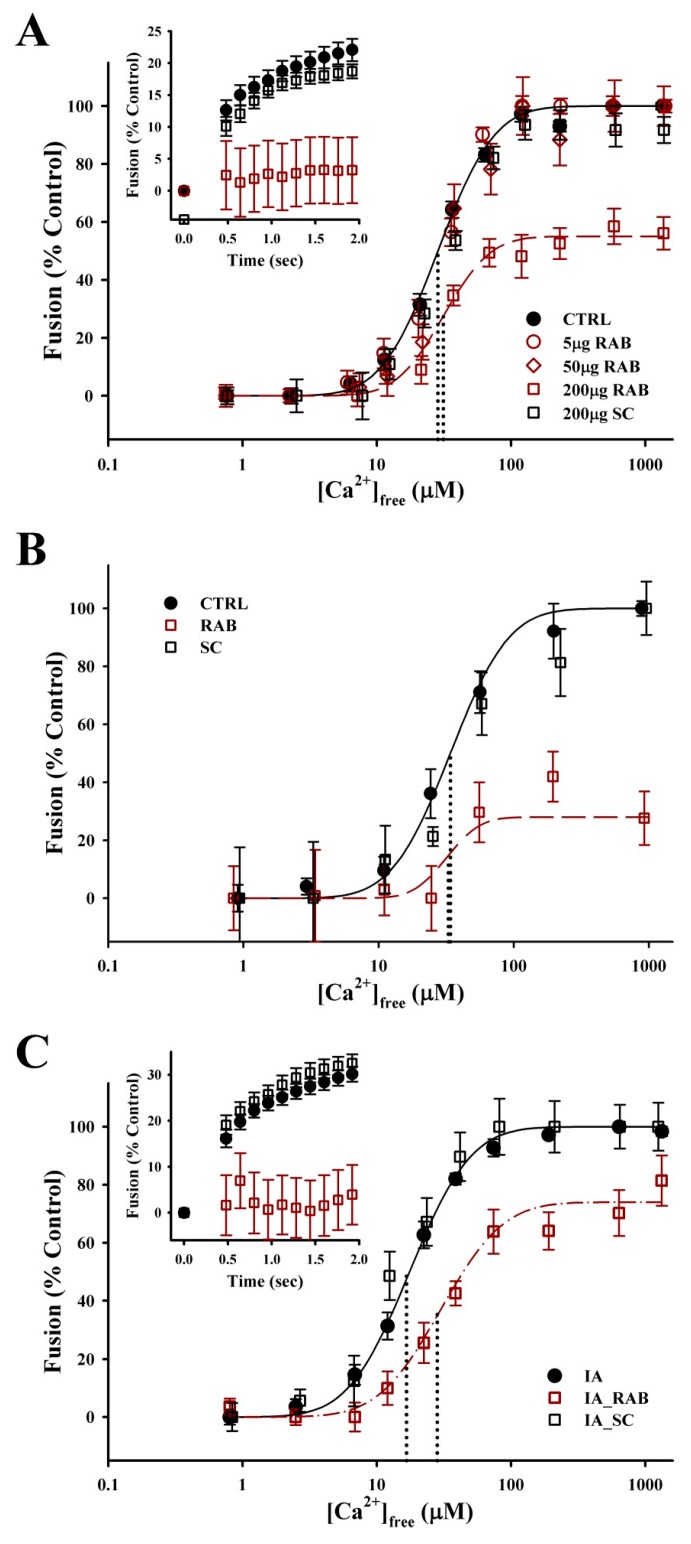
Rab-GTPase modulates the efficiency of Ca^2+^-triggered exocytosis. (**A**) Ca^2+^ activity curves for the standard CV–CV fusion assay supplemented with 100 µM GTPγS (CTRL; *n* = 11), plus the addition of 5 µg/mL RAB (*n* = 5), 50 µg/mL RAB (*n* = 5), and 200 µg/mL RAB (*n* = 6) or 200 µg/mL scrambled (SC; *n* = 4) peptide for 30 min. Fusion kinetics (inset; *n* = 4–9) in response to 41.8 ± 3.5 µM [Ca^2+^]_free_ shown for CTRL, 200 µg/mL RAB and 200 µg/mL SC peptide. (**B**) Ca^2+^ activity curves for the modified settle assay supplemented with 100 µM GTPγS (CTRL; *n* = 3), plus the addition of 200 µg/mL RAB (*n* = 2) or 200 µg/mL SC (*n* = 2) peptide for 30 min. (**C**) Ca^2+^ activity curves for standard CV–CV fusion assay pre-treated with 20 mM IA for 20 min. IA treatment was followed by a second incubation in fresh BIM supplemented with 100 µM GTPγS (IA control; *n* = 5), plus the addition of 200 µg/mL RAB (*n* = 5) or 200 µg/mL SC (*n* = 3) peptide for 30 min. Fusion kinetics (inset; *n* = 3–4) in response to 45.4 ± 6.2 µM [Ca^2+^]_free_ are shown.

To directly investigate the role of Rab-GTPases in modulating the late steps of Ca^2+^-triggered exocytosis, an exogenous RAB3 effector domain peptide was used [47]; this peptide contains the G2- box/Rab Family 1 motif which shares 50%–67% identity (67%–100% similarity) with the *S. purpuratus* Rab GTPases identified (Table 2). The addition of the RAB effector binding domain, in the presence of GTPγS, resulted in a concentration-dependent inhibition of CV–CV fusion (Figure 6A). Treatment with 200 µg/mL RAB peptide for 30 min decreased the extent of fusion to 54.0 ± 9.1% (*n* = 6; *p* ≤ 0.001), but no effect on Ca^2+^ sensitivity was observed (control EC_50_ = 32.1 ± 2.4 µm; 200 µg/mL RAB EC_50_ = 36.6 ± 2.3 µm). The initial fusion kinetics were also inhibited in response to 41.8 ± 3.5 µm [Ca^2+^]_free_ (*n* = 6; *p* ≤ 0.05). To determine whether the effect of the RAB peptide was, in part, due to modulation of vesicle priming/docking, a modified fusion assay in which CVs were allowed to settle into contact was also carried out (Figure 6B) [3,6,30,39]. In the settle assay, treatment with 200 µg/mL RAB peptide for 30 min decreased the extent of CV–CV fusion to 37.0% ± 11.7% (*n* = 2; *p* ≤ 0.05). As with the standard fusion assay, there was no effect on Ca^2+^ sensitivity (Control EC_50_ = 40.7 ± 13.7 µm; 200 µg/mL RAB EC_50_ = 39.4 ± 10.8 µm). The addition of 200 µg/mL scrambled peptide had no effect on the Ca^2+^ sensitivity, kinetics or extent of fusion in either the standard or settle assays.

While no effect on Ca^2+^-sensitivity was observed with the addition of RAB peptide alone, we tested for an interaction between thiol treatment and Rab GTPase function. CVs were pretreated with 20 mM IA for 20 min which resulted in a typical leftward shift in EC_50_ (Figure 6C). Following the washout with IA, CVs were treated with 200 µg/mL of the RAB effector domain peptide or 200 µg/mL of the scrambled peptide, in the presence of GTPγS, for 30 min. Treatment with the RAB peptide shifted the EC_50_ rightward back toward that of naïve, untreated CV (IA EC_50_ = 16.7 ± 2.6 µm versus IA_ RAB EC_50_ = 28.2 ± 5.3 µm; *n* = 5; *p* = 0.0865). The extent of fusion in CV pretreated with IA followed by treatment with RAB peptide was 74.6% ± 19.2%. Initial fusion kinetics were also inhibited in response to 48.5 ± 6.6 µm [Ca^2+^]_free_ (*n* = 4; *p* ≤ 0.05). The Ca^2+^ sensitivity, kinetics or extent of fusion in CV pretreated with IA followed by treatment with scrambled peptide did not differ from the IA pretreatment condition.

## 4. Discussion

Thiol-reactive agents are powerful tools in an unbiased approach to investigating proteoform functions in regulated exocytosis [35,37,38,39,40,45]. However, as these reagents are also non-selective, promiscuously labeling free sulfhydryl groups on multiple proteins, several strategies were taken to reduce the complexity of the labeled proteome. First, isolation of native secretory vesicles from urchin oocytes provided a stage-specific preparation for studying the late steps of Ca^2+^-triggered exocytosis. Second, several fluorescent thiol reagents that potentiate the Ca^2+^ sensitivity and kinetics of CV–CV fusion were characterized and employed as ‘functional’ tags to label CV membrane proteoforms involved. Third, prefractionation of total CV membrane or cholesterol-enriched CV membrane, followed by high-resolution 2DE, was carried out to target downstream mass spectrometric analyses on the identification of reproducibly labeled, secretory vesicle membrane proteoforms. This rigorous approach using a top-down proteomic methodology identified several viable proteoform candidates, and validation studies suggest multiple roles for Rab-GTPases in modulating the efficiency of exocytosis.

The urchin CV model system provides an in vitro preparation of native secretory vesicles amenable to tightly coupled functional and molecular analyses [22,33,34]. CVs and other secretory vesicles have similar lipid compositions [9,16,17] and contain homologues of the major membrane trafficking proteins [18,30,31,32]. Isolated CVs retain all the critical cellular components to support fast, Ca^2+^-triggered exocytosis [33,35]. Furthermore, unlike other types of secretory vesicles, isolated CVs remain primed and fusion competent, requiring only an increase in [Ca^2+^]_free_ to trigger membrane fusion [35,53]. With ~15,000 CVs per oocyte [33], ample material can be obtained for both functional and proteomic analysis from a single preparation. The removal of other cellular components, including the cytosol and PM, minimizes ‘background’ proteins in the proteome, enabling targeted studies on the molecular mechanisms underlying regulated exocytosis.

Thiol-reactive reagents have a biphasic effect on the Ca^2+^-triggering steps of exocytosis [39,40]. Many of these reagents (i.e., NEM) inhibit CV fusion at low millimolar concentrations [30,35,36,37,38,39,40]; on the other hand, IA potentiates at low millimolar concentrations and inhibits at high millimolar concentrations [39]. The ability of thiol reagents to potentiate or inhibit the Ca^2+^-triggering steps of exocytosis appears to be dependent of their hydrophobicity (Figure S1). Addition of a fluorescein tag (IAF) results in more potent inhibition, whereas a lucifer yellow tag (LYIA) with charged sulfate groups retains the ability to enhance Ca^2+^ sensitivity [39,40]. Given that bromobimanes have similar molecular structures to iodoacetamides, both in terms of the thiol-reactive halogen moieties and the polar nature of the oxygen and iodide/bromide groups, it was likely that they would exhibit similar effects. Treatment with these fluorescent thiol reagents resulted in average leftward shifts in Ca^2+^ sensitivity in the range of 5–10 µM [Ca^2+^]_free_ ― LYIA ∆pCa = −0.25 ± 0.05, mBB ∆pCa = −0.18 ± 0.06 and dBB ∆pCa = −0.27 ± 0.04 ― which is of comparable magnitude to the previously observed effects of IA (∆pCa = −0.27 ± 0.06 [39]). Furthermore, these reagents had an even more profound effect on Sr^2+^-triggered fusion, resulting in average leftward shifts in Sr^2+^ sensitivity in the range of 1–2 mM ― LYIA ∆pMe = −0.40 ± 0.8, mBB ∆pMe = −0.38 ± 0.10 and dBB ∆pMe = −0.40 ± 0.05. It has been suggested that Ca^2+^ mimetics, such as Sr^2+^, retain the ability to trigger membrane fusion, but have a lower affinity for those Me^2+^ binding sites that regulate the efficiency exocytosis [30,39]. The data suggest that all three of these fluorescent reagents act on common thiol sites not directly involved in the membrane fusion mechanism *per se*, but that modulate the Ca^2+^ sensitivity of the late steps of exocytosis.

These fluorescent thiol-reactive reagents enhance the Ca^2+^ sensitivity and kinetics of exocytosis and concomitantly labeled candidate CV membrane proteoforms, mediating these functional effects. In total, thirty-eight proteoforms were identified from fluorescently labeled spots in 2DE gels: twenty-one from the total CV membrane proteome, eleven from the cholesterol-enriched CV membrane proteome, and six from both proteomes. Numerous high abundance, high molecular weight proteins were found to correspond to CV and/or yolk granule content proteins (e.g., apolipoprotein, rendezvin, major yolk protein and vitellogenin; data not shown). This was not entirely unexpected, as these vesicles contain many glycoproteins that undergo extensive crosslinking to form the fertilization envelope [54,55], which would retain strong interactions with the CV membrane during isolation. Although we were able to identify co-resolving proteoforms (e.g., long-chain-fatty-acid- CoA ligase 1), the presence of these high abundance content proteins may well have interfered to some extent with the identification of lower abundance CV membrane proteoforms. The optimizations of 2DE workflows to improve in the resolution and detection of low abundance CV membrane proteoforms, including the prefractionation of membrane isolates, transfer of proteins to PVDF membrane to improve signal from fluorescent labels, separation on large format IPG strips, third dimension separations and pooling of spots from replicate gels, were thus employed. Despite these efforts, seven of the labeled spots, as well as numerous areas of the peptide front, yielded no significant database matches. Although the genome of *S. purpuratus* has been sequenced [56], there is still limited annotation and under-representation of the entire proteome. Many of the proteoforms identified are predicted based on genome sequences, and therefore have little experimental evidence at the protein (i.e., amino acid sequence) level so far.

Of the labeled proteoforms identified, approximately half were associated with the endomembrane system (~55%) and half were metabolic enzymes (~47%) that are typically associated with mitochondria (i.e., canonical protein association), with a small amount of overlap (~10%). This raises the question of whether other organelles (or fragments of these organelles) co-purify or whether these metabolic enzymes are actually located on (or within) secretory vesicles; the purity of these well-established CV preparations at the level of electron microscopy [54] suggest the latter. Indeed, resident mitochondrial and endoplasmic reticulum (ER) proteins are commonly identified in other purified secretory vesicle preparations [16,19]. While a minimal amount of contamination from other organelles would be expected, the abundance of these metabolic enzymes seems more indicative of a local function on secretory vesicles. A recent large-scale proteomic study of human cell lines revealed that over 50% of proteins map to more than one subcellular location [57]; thus, the use of top-down analytical approaches, such as high-resolution 2DE, to resolve and identify proteoforms, is critical to genuinely understand molecular mechanisms and cellular processes [41,58,59]. The distribution of protein species across compartments in the endomembrane system would be a predictable consequence of transport vesicles budding from one site and being delivered to another. This would certainly be the case with Rab-GTPases, which play an important role in targeting and tethering vesicles from one compartment to the next [60]. For instance, during oocyte maturation, CVs are produced by the fusion of smaller transport vesicles, and then move to their docking/fusion sites at the PM. Other known vesicular membrane proteins identified were two subunits of the V-type ATPase involved in proton transport and two CV proteins (18 kDa and 34 kDa) for which functions are largely uncharacterized. In addition, cytoskeletal proteoforms were also identified.

Two major cytoskeletal components, actin and tubulin, were both found to be labeled. The cytoskeleton is important for vesicle mobilization and transport [61,62,63,64,65], but additional roles in later stages of regulated secretion have also been proposed. A dense cortical actin network is closely associated with release sites in secretory cells [62], and both actin and tubulin are isolated with the membranes of secretory vesicles [16,19,49]. Pharmacological disruption of actin filaments has been shown to enhance the efficiency of exocytosis, suggesting that the cytoskeleton acts as a barrier to secretion [66], which is supported by imaging of actin remodeling at vesicular release sites [61,67,68]. This is consistent with experiments using the CV model in which pharmacological treatments with actin or tubulin stabilizing and destabilizing reagents, or the addition of exogenous cytoskeletal components, do not alter the Ca^2+^-sensitivity, kinetics or extent of fusion [49] (Figure S6). These data indicate that actin and tubulin act at upstream stages of exocytosis (which, fully-docked and release-ready CV have already passed through), and are not minimally essential to the fundamental Ca^2+^-triggering and membrane merger steps. The cystoskeleton may, nonetheless, modulate these late steps of exocytosis in intact cells by either organizing critical membrane components at release sites and/or exerting tension to facilitate fusion pore expansion [62,63], but, as previously demonstrated with CV, are not essential to the Ca^2+^-triggered membrane fusion mechanism *per se* [49].

The largest number of thiol-labeled CV membrane proteoforms belong to the Rab family of GTPases, which have well-characterized roles in vesicular trafficking [60,69,70,71]. Rab proteins associate with cholesterol-enriched membrane domains through their prenylation anchors [72]. Rabs cycle between membrane-associated and cytosolic distributions in their GTP and GDP-bound states which are regulated by nucleotide exchange factors (GEFs) and GTPase activating proteins (GAPs), respectively. When Rabs are delivered to the membrane by GDP-dissociation inhibitors (GDIs), GDIs are removed by GDI-displacement factors (GDFs) and subsequently GDP is converted to GTP by GEFs. In the active GTP-bound form, Rabs recruit effector proteins, including a myriad of sorting adaptors, tethering factors, motor proteins, kinases, phosphatases, Ca^2+^/phospholipid binding proteins and other GEFs/GAPs [69,70,71]. Thus, it is postulated that a cascade of Rab- dependent interactions coordinate the translocation of vesicles throughout the cell [60]. Different Rab GTPases localize to select ‘Rab’ domains on distinct intracellular organelles, providing a level of specificity for targeting and tethering transport vesicles to various cellular compartments [69]. Yet, the number of Rab GTPases associated with membranes of isolated CVs (at least eight Rabs—Rab1, Rab2, Rab5, Rab7, Rab11, Rab13, Rab33 – Rab3 [47]), insulin granules (14 Rabs [73]) and synaptic vesicles (>20 Rabs [16,74]) is indicative of overlapping localization, if not also a redundancy in function. Furthermore, a single Rab GTPase may regulate the function of several effector proteins, and in turn, a single effector protein may be regulated by multiple Rabs [69,70].

To this end, we further explored the role of Rab GTPases in regulated exocytosis in the urchin CV model. An exogenous RAB peptide, containing the effector domain of Rab3 [47], was found to inhibit CV–CV fusion. This Rab family 1 motif is largely conserved (>50%) among the Rab GTPases and across different species (Table 2), and this effector domain peptide has been previously shown to interfere with the formation of the fertilization envelope in urchin oocytes [47]. An addition of exogenous RAB peptide inhibited the extent of fusion by ~63% in the modified settle assay, compared to ~46% in the standard assay; this strongly indicates an effect on upstream stages of CV tethering/docking/priming [1,2,3,6,8,30,39]. With the addition of the RAB effector domain, there was no effect on Ca^2+^-sensitivity; however, after pretreatment with IA, treatment with the RAB peptide resulted in a decrease in Ca^2+^-sensitivity, mitigating the potentiation effect of IA. Previously, we postulated that thiol reagents may enhance the Ca^2+^-sensing steps of regulated secretion by either directly acting on a Ca^2+^-binding protein or by acting on an accessory protein that interacts with the Ca^2+^-binding protein and/or other components of the exocytic machinery [39]. In this scenario, we speculate that Rab GTPases may continue to interact with proteins at vesicular release sites subsequent to the completion of tethering and docking. Treatment with thiol reagents would block this interaction, releasing the Rab effector and allowing the later steps of Ca^2+^-triggered membrane fusion to proceed unhindered. This would be consistent with observations that an increase in [Ca^2+^]_free_ can partially overcome the inhibition of fertilization envelope formation by exogenous RAB peptide in intact oocytes [47]. Furthermore, the data are consistent with evidence that Rab3 acts through a negative, clamp-like mechanism to regulate the late steps of exocytosis in neuronal [75] and neuroendocrine [76,77] cells.

These data highlight the dual role of specific proteoforms in exocytosis, acting as both positive and negative modulators of regulated secretion, depending on the steps of the pathway being investigated. Several Rab GTPases are associated with secretory vesicles: Rab1, Rab2, Rab3, Rab4, Rab5, Rab7, Rab10, Rab11, Rab14 and Rab27 all co-purify (i.e., enrichment) during the isolation of synaptic vesicles [74], and we have found several of these to be thiol-labeled in the CV membrane proteome. Rab3 and Rab 27 have gained the most interest in Ca^2+^-triggered exocytosis [70]; however, Rab11 and Rab4 have been shown to regulate Ca^2+^-dependent secretion in (neuro)endocrine cells [78,79] and platelets [80], respectively. While Rab proteins have critical upstream roles in vesicle targeting, tethering and docking [60,69,70,71], they also appear to have modulatory effects on the efficiency of exocytosis, even the fusion of fully release-ready vesicles. Rabs have been shown to modulate SNARE function, both through interactions with SM proteins [81,82,83] and via their other effectors [84,85,86,87,88]. This suggests that disassociation of the Rab/effector complex from the SNARE complex is required for vesicles to reach a fully docked and primed state [88]. Additionally, many Rab effectors, such as Rabphilin and synaptotagmin-like proteins (Slp1-5), contain C2A/C2B domains capable of binding Ca^2+^ and phospholipids [70] which may mediate any additional SNARE-independent modulation of exocytosis. Rab effectors have also been shown to regulate phosphoinositide conversion [89,90], which has been previously shown to modulate the efficiency of CV–CV fusion, possibly by regulating late priming steps [6,7]. Further elucidation of the function of each Rab GTPase, and their selective or non-selective effectors, will shed additional light on the regulatory mechanisms of the late steps of regulated exocytosis. The ability of Rabs, and other upstream exocytotic proteins, to modulate the efficiency of Ca^2+^-triggered membrane fusion increases the complexity of control over this tightly regulated process, allowing for graded responses to different external stimuli.

This does not preclude other thiol-labeled proteoforms from also contributing to efficiency of regulated exocytosis. The presence of several metabolic enzymes on secretory vesicles suggests local regulatory mechanisms to ensure optimal lipid and protein function. Along with cholesterol- and sphingomyelin-enriched membrane domains [8,9,10,11,45,46], other lipid species, such as phosphoinositides, participate in the organization of the exocytotic machinery at release sites [4]. The generation of phospholipid metabolites from phospholipase D [3] and phospholipase A [2] can also modulate the efficiency of exocytosis via roles in upstream stages, such as docking/priming. Here, we have identified two labeled enzymes involved in lipid metabolism, long-chain-fatty-acid-CoA ligase 1 and long-chain specific acyl-CoA dehydrogenase, which may have roles in regulating local fatty acid metabolism in secretory vesicles. Long chain acyl-CoA has been shown to enhance secretion in pancreatic beta-cells, perhaps through direct interaction with exocytotic proteins [91]. Furthermore, several proteoforms with chaperone functions were labeled, including calreticulin, calcistorin, disulfide-isomerase 2-like, protein disulfide-isomerase A3, 60 kDa heat shock protein and elongation factor Tu. While some may be involved in packaging of content into CV (ie., calreticulin), others may interact with proteins involved in exocytosis. Numerous protein chaperones operate in nerve terminals to maintain the proper conformation and function of proteins at different steps of the exocytotic pathway [92,93,94]. These thiol-labeled proteoforms, along with uncharacterized or unidentified proteins from our study, warrant further investigation. As our understanding of the molecular mechanisms underlying different steps of Ca^2+^-triggered exocytosis evolves, it becomes increasingly apparent that the coordinated action of a multitude of proteins and lipids are required for fast, efficient cellular secretion, and that many aspects of exocytotic pathway are very highly conserved.

## 5. Conclusions

In this study, we have taken a top-down proteomic approach to studying the molecular mechanisms underlying the late steps of Ca^2+^-triggered exocytosis. Thiol-reactive reagents were employed as functional tags to label candidate proteoforms that regulated the Ca^2+^-sensitivity and the kinetics of secretory vesicle (i.e., CV) fusion. A targeted experimental paradigm, including subcellular fractionation and optimized 2DE resolution of labeled proteoforms, yielded a targeted list of proteins for further investigation. While a majority evidence suggests Rab GTPases have roles upstream in tethering/docking, the data here are unique in also indicating a role in later steps that modulate Ca^2+^-sensitivity. Furthermore, several other labeled proteoforms may have additional roles that ensure optimal function of the exocytotic machinery (both proteins and lipids) in regulated secretion.

## Figures and Tables

**Table 1:** Thiol-labelled protein spots identified from total and cholesterol-enriched CV membrane proteomes.

Spot #	Accession #	Protein Description	Organism	Total CVmem		Chol- CVmem		Theoretical		Experimental ^c^		Biological Function ^d,e^
				Mascot ^b^	Peptides	Mascot _b_	Peptides	pI	MW	pI	MW	
1		unidentified		n/s	-					5.3	85.4	
2	XP_011662952.1	succinate dehydrogenase, flavoprotein subunit ^a^	*S. purpuratus*	299	5			5.15	61.5	5.3	84.9	metabolic process
3	XP_011662952.1	succinate dehydrogenase, flavoprotein subunit ^a^	*S. purpuratus*	137	3			5.15	61.5	5.4	84.9	metabolic process
4		unidentified		n/s	-					5.7	84.2	
5		unidentified		n/s	-					5.8	83.0	
6	XP_779941.1	long-chain-fatty-acid--CoA ligase 1 ^a^	*S. purpuratus*	201	3	195	6	5.58	73.5	5.9	82.4	metabolic process
7	NP_999643.1	calreticulin precursor	*S. purpuratus*	303	7	116	2	4.57	95.5	3.9	65.0	protein folding
	XP_782447.2	dihydrolipoyl dehydrogenase ^a^	*S. purpuratus*	111	2			7.10	101.3			metabolic process
8		NP_999697.1	*S. purpuratus*	257	3			4.38	54.9	4.1	68.1	redox/protein folding
8a	NP_999697.1	ER calcistorin precursor	*S. purpuratus*			126	3	4.38	54.9	4.7	71.1	redox/protein folding
	XP_795205.2	60 kDa heat shock protein ^a^	*S. purpuratus*			119	3	5.24	62.2			protein folding
	XP_011677933.1	disulfide-isomerase 2-like ^a^	*S. purpuratus*			114	3	5.14	45.8			redox/protein folding
8b	NP_999697.1	ER calcistorin precursor	*S. purpuratus*			220	6	4.57	95.5	5.0	71.1	redox/protein folding
	XP_779941.1	long-chain-fatty-acid--CoA ligase 1 ^a^	*S. purpuratus*			60	2	5.58	73. 5			metabolic process
9	XP_795205.2	60 kDa heat shock protein ^a^	*S. purpuratus*	685	9			5.24	62.2	4.9	70.4	protein folding
10	XP_795205.2	60 kDa heat shock protein ^a^	*S. purpuratus*	612	7			5.24	62.2	5.0	70.7	protein folding
11	XP_003726658.1	V-type proton ATPase subunit A isoform X4 ^a^	*S. purpuratus*	131	3	561	16	5.32	67.9	5.4	75.5	ion transport
12	XP_003726658.1	V-type proton ATPase subunit A isoform X4 ^a^	*S. purpuratus*	183	3			5.32	67.9	5.5	75.3	ion transport
13		unidentified		n/s	-					5.5	71.5	
14	XP_791790.1	tubulin beta chain ^a^	*S. purpuratus*	443	9			4.73	50.1	4.8	62.1	cellular organization
15	XP_789821.1	tubulin beta chain ^a^	*S. purpuratus*	172	4			4.61	50.1	5.0	61.8	cellular organization
	XP_003731782.2	aldehyde dehydrogenase ^a^	*S. purpuratus*	597	13			5.32	41.2			metabolic process
15b	XP_011662394.1	V-type proton ATPase subunit B isoform X1 ^a^	*S. purpuratus*			341	8	5.21	55.1	5.5	64.6	ion transport
	AFG26286.1	aldolase class-1 protein	*A. japonicus*			82	2	8.30	39.4			metabolic process
15c	XP_011661972.1	protein disulfide-isomerase A3 ^a^	*S. purpuratus*			184	6	5.44	65.5	5.5	60.8	redox/protein folding
16				n/s	-					4.6	57.3	
17	NP_001116974.1	ATP synthase beta subunit	*S. purpuratus*	1084	13			5.14	56.0	4.6	55.1	metabolic process
	Q25117.1	ATP synthase subunit beta	*H. pulcherrimus*			249	6	5.10	56.1			metabolic process
	XP_786080.3	arginine kinase ^a^	*S. purpuratus*			76	2	5.26	46.5			signal transduction
18	XP_786753.1	succinyl-CoA ligase subunit beta ^a^	*S. purpuratus*	213	4			5.16	49.1	4.7	49.3	metabolic process
19		unidentified		n/s	-					5.1	48.3	
20	XP_782503.3	long-chain specific acyl-CoA dehydrogenase, isoform X1 ^a^	*S. purpuratus*	130	5			5.67	48.9	5.2	47.7	metabolic process
21	XP_003725373.1	actin, cytoskeletal 3 B ^a^	*S. purpuratus*	112	3			5.22	41.8	5.3	46.9	cellular organization
22	XP_786922.3	NADH dehydrogenase, iron-sulfur protein 2 ^a^	*S. purpuratus*	123	3			6.0	52.7	5.8	51.9	metabolic process
23	XP_788937.1	elongation factor Tu ^a^	*S. purpuratus*	333	5			6.3	50.0	5.9	49.3	protein biosynthesis
												/protein folding
24	XP_011665962.1	isocitrate dehydrogenase [NADP], isoform X1 ^a^	*S. purpuratus*	112	4			6.2	50.2	5.9	45.7	metabolic process
25	XP_011665962.1	isocitrate dehydrogenase [NADP], isoform X1 ^a^	*S. purpuratus*	305	5			6.2	50.2	6.0	45.2	metabolic process
26	XP_011665962.1	isocitrate dehydrogenase [NADP], isoform X1 ^a^	*S. purpuratus*	120	4			6.2	50.2	6.1	45.2	metabolic process
27	XP_789891.3	cytochrome b-c1 complex subunit 2 ^a^	*S. purpuratus*	76	3			8.7	50.8	6.8	45.5	metabolic process
28	AAG15425.1	34kDa cortical vesicle protein, partial	*S. purpuratus*	158	2			5.2	30.7	4.2	26.0	uncharacterized
29		unidentified		n/s	-					5.3	25.8	
30	XP_003725895.1	electron transfer flavoprotein subunit beta isoform X1 ^a^	*S. purpuratus*	174	5			5.5	27.8	5.5	27.6	metabolic process
31	XP_780266.1	voltage-dependent anion-selective channel protein 2 ^a^	*S. purpuratus*	227	3			6.3	30.4	6.0	27.6	ion transport
32		unidentified		n/s	-					3.2	21.8	
32a	NP_001116984.1	rab11 GTPase homolog SUrab11	*S. purpuratus*			107	3	6.1	24.5	3.6	22.2	vesicular trafficking
32b	NP_001116983.1	rab7 GTPase homolog SUrab7	*S. purpuratus*			166	4	5.5	23.1	3.5	20.3	vesicular trafficking
	XP_783878.1	ras-related protein Rab-5B ^a^	*S. purpuratus*			127	4	8.3	23.6			vesicular trafficking
	XP_001201172.2	ADP-ribosylation factor-like protein 8B-A ^a^	*S. purpuratus*			120	3	6.8	21.6			vesicular trafficking
32c	XP_782537.1	ras-related protein Rab-2A isoform X1 ^a^	*S. purpuratus*			91	2	6.2	23.7	4.6	23.3	vesicular trafficking
32d						n/s	-			4.5	22.1	
32e	NP_001116983.1	rab7 GTPase homolog SUrab7	*S. purpuratus*			234	6	5.5	23.1	4.4	20.3	vesicular trafficking
	PSN32081.1	ras-related protein Rab-2A	*B.germanica*			116	3	6.0	23.6			vesicular trafficking
33	NP_999641.1	18kDa egg cortical vesicle protein precursor	*S. purpuratus*	208	2			4.8	20.6	4.1	12.7	uncharacterized
34	NP_999641.1	18kDa egg cortical vesicle protein precursor cytochrome c oxidase subunit 5A ^a^	*S. purpuratus*	248	3			4.8	20.6	4.5	12.6	uncharacterized
	XP_784558.1		*S. purpuratus*	167	2			5.3	16.9			metabolic process
35	ABO26625.1	Ras-related protein Rab-1A	*H. discus discus*	73	3			5.6	22.8	4.2	10.4	vesicular trafficking
	XP_015905011.1	ras-related protein Rab-33B	*P.tepidariorum*	72	2			7.6	25.6			vesicular trafficking
	XP_020901910.1	ras-related protein Rab-13	*E. pallida*			82	2	7.6	24.3			vesicular trafficking
36	XP_786378.1	cytochrome c oxidase subunit 5B ^a^	*S. purpuratus*	231	5			8.3	14.2	6.8	11.4	metabolic process
37	XP_794003.1	succinate dehydrogenase, iron-sulfur subunit ^a^	*S. purpuratus*	145	4			8.8	31.8	9.7	24.8	metabolic process

^a^ Predicted from genome sequence. ^b^ If protein identification occurred from the same spot across multiple gels, the highest probability match was reported. ^c^ Experimental pI and MW (kDa) estimated from total CV membrane proteome (large IPG), unless only observed in cholesterol-enriched CV membrane proteome (mini IPG). ^d^ If protein information in the UniprotKB database on *Strongylocentrotus purpuratus* was limited, biological function from mammalian (human) homologue were reported. ^e^ Biological function representative of GO biological processes in broadest terms.

**Table 2:** Conserved sequence of RAB peptide effector domain.

	G2-Box/RabF1 Motif	*S. purparatus* ^a^		*H. sapiens* ^b^
		% Identity	% Similarity	% Identity
**Rab3**	TVGIDF	100	100	100
**Rab2**	TIGVEF	50	100	100
**Rab5**	TIGAAF	50	67	100
**Rab7**	TIGADF	67	83	100
**Rab11**	TIGVEF	50	100	100

^**a**^ Conserved sequence identity and similarity reported relative to Rab 3 (*S. purparatus*). ^**b**^ Conserved sequence identity reported relative to *S. purparatus.*

## Supplementary Materials

The following are available online at https://www.mdpi.com/2227-7382/7/4/34/s1, Figure S1: Chemical structures of common thiol-reactive reagents; Figure S2: Inhibition of Ca^2+^-triggered exocytosis with fluorescent thiol reagents; Figure S3: Soluble proteome of CV treated with fluorescent thiol reagents; Figure S4: Cholesterol- enriched membrane proteome of CV treated with fluorescent thiol reagents; Figure S5: Comparison of total CV membrane and cholesterol-enriched CV membrane proteomes; Figure S6: Tubulin does not regulate the efficiency of Ca^2+^-triggered exocytosis.
