# Supplementary material for: Unbiased Thiol-Labeling and Top-Down Proteomic Analyses Implicate Multiple Proteins in the Late Steps of Regulated Secretion
Source: Proteomes. 2019 Sep 27;7(4):34. doi: 10.3390/proteomes7040034 (PMC6958363; doi:10.3390/proteomes7040034)
Supplement: Supplementary file 1 [file proteomes-07-00034-s001.pdf]

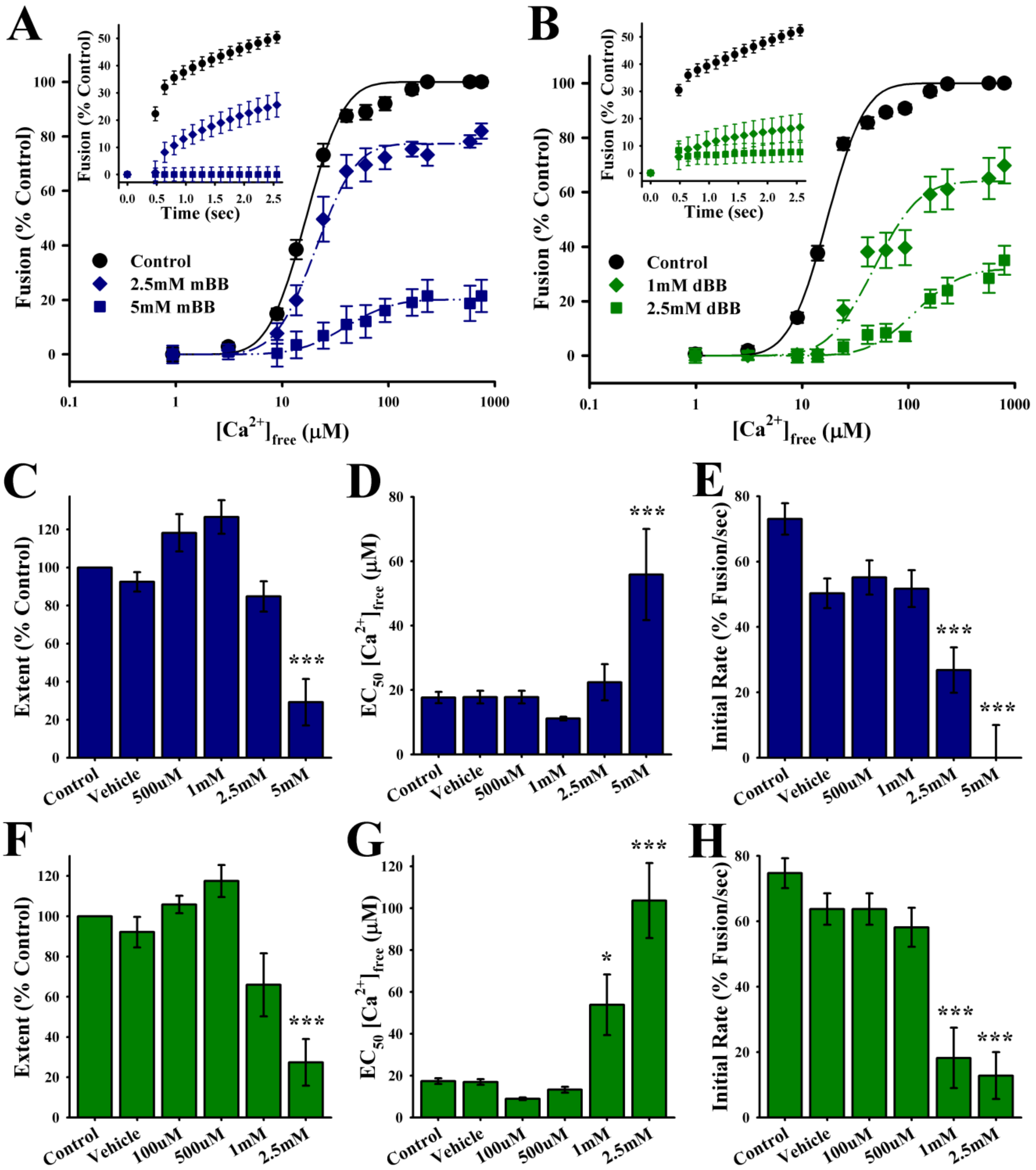

Figure S1: Inhibition of Ca^{2+}-triggered exocytosis with fluorescent thiol reagents.

(**A**) Ca^{2+} activity curves (n = 4) and fusion kinetics (n=4; inset) for standard CV-CV fusion assay after treatment with 2.5mM and 5mM mBB for 1h at 25°C. (**B**) Ca^{2+} activity curves (n = 4) and fusion kinetics (n=4; inset) for standard CV-CV fusion assay after treatment with 1mM and 2.5mM dBB for 1h at 25°C. Summary of concentration-dependent effects of mBB (**C**-**E**) and dBB (**F**-**H**) treatment on the extent of fusion (**C** and **F**), Ca^{2+}-sensitivity (**D** and **G**) and initial rate of kinetics (**E** and **H**). Data presented as mean ± SEM; statistical analysis by one-way ANOVA with Bonferroni multiple comparison test versus control (*p<0.05, **p<0.01, ***p<0.001).

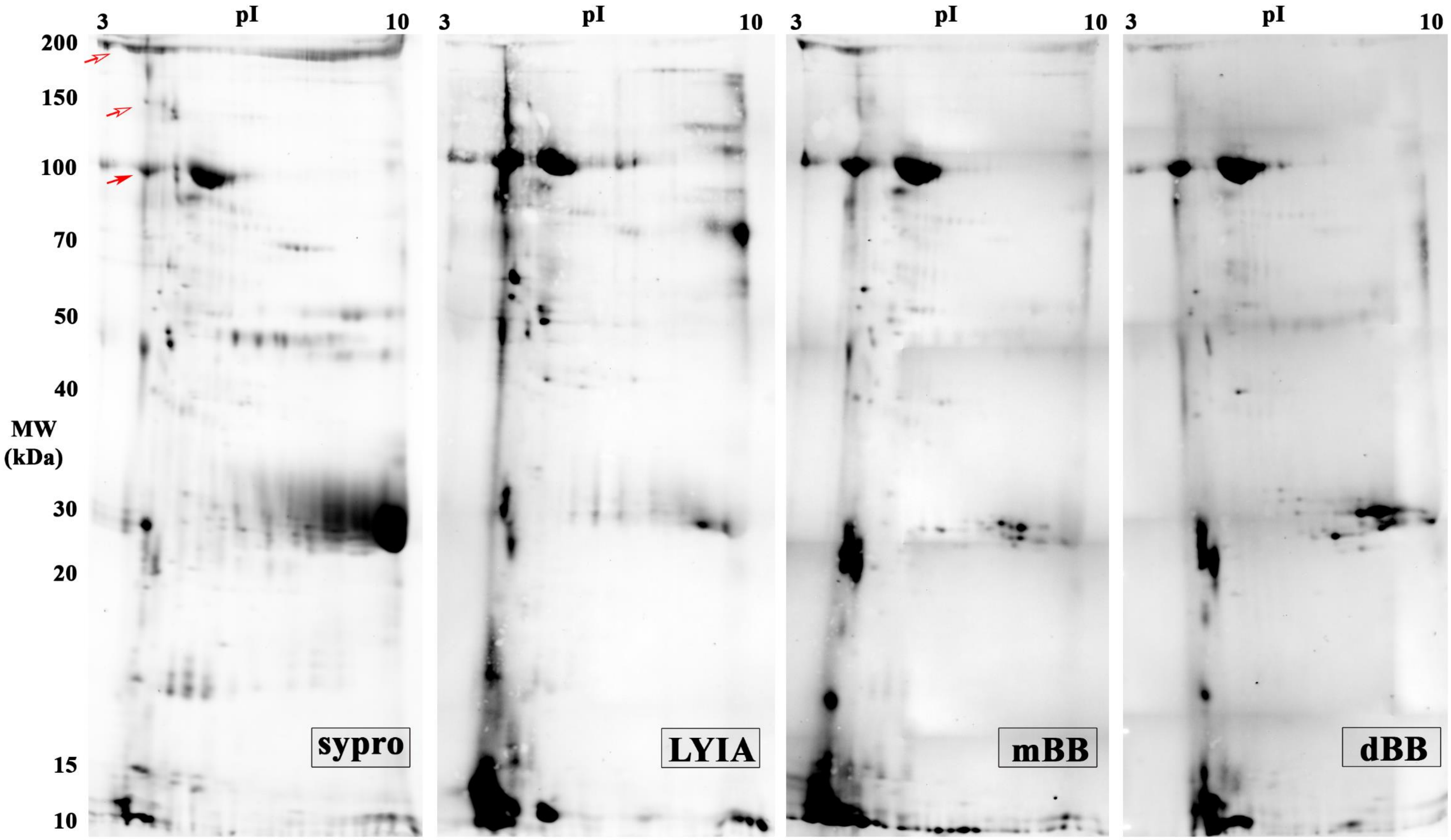

Figure S2: Soluble proteome of CV treated with fluorescent thiol reagents.

(**A**) Representative 2DE gel image of soluble membrane proteome from untreated CV resolved by mini 3-10NL IPG and large 10-14% SDS-PAGE format, stained for total protein with Sypro Ruby (n=2). Representative PVDF blots images (n=2) scanned for LYIA (**B**), mBB (**C**) and dBB (**D**). Open and closed arrows indicate high molecular weight CV content proteins observed in CV membrane proteomes.

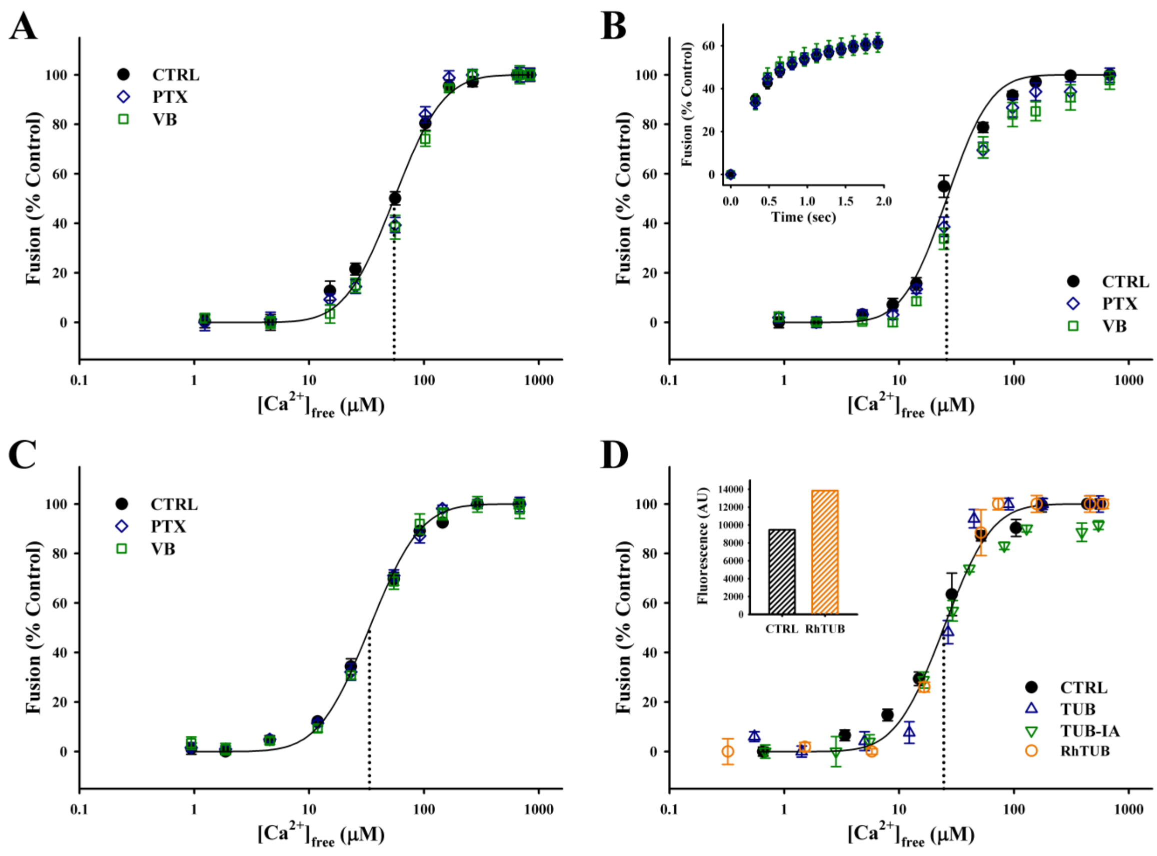

Figure S3: Tubulin does not regulate the efficiency of exocytosis.

Ca^{2+}-activity curves standard CV-PM fusion assay (**A**; n=2), standard CV-CV fusion assay (**B**; n=4-5) and modified CV-CV settle assay (**C**; n=3) in the presence tubulin destabilizing (10μM vinblastine - VB) and polymerizing (10μM paclitaxel - PTX) reagents following 1h incubation at 25°C. Fusion kinetics (**B;** inset) in response to 139.7 ± 17.1μM $[Ca^{2+}]_{free}$ for the same treatment conditions. Ca^{2+}-activity curves for isolated CV treated with either 200μg exogenous tubulin, 100μg exogenous rhodamine-tubulin or 200μg exogenous tubulin pretreated with IA in the presence of taxol and GTP to promote polymerization (**D**; n=1). Following 1h incubation at 25°C, excess reagent was removed by centrifugation prior to standard CV-CV fusion assays. An increase in fluorescence of vesicles treated with rhodamine-tubulin indicates the exogenous bovine tubulin polymerized with urchin CV tubulin (**D; inset**).
